# Biopsychosocial interplay of personal injury insurance, legal frameworks, and functional neurological disorder: an emerging perspective on recovery and disability determinants

**DOI:** 10.3389/fneur.2026.1904281

**Published:** 2026-10-01

**Authors:** Edwina Rose Sekine, Sarah Oxford, Shannon Gray, Ross Iles, Janine McMillan, Richard Kanaan

**Affiliations:** 1 Healthy Working Lives Research Group, School of Public Health and Preventive Medicine, Monash University, Melbourne, VIC, Australia; 2 Institute for Safety, Compensation and Recovery Research, Monash University, Melbourne, VIC, Australia; 3 Department of Psychiatry, University of Melbourne, Austin Health, Heidelberg, VIC, Australia

**Keywords:** compensation, disability, functional neurological disorder, iatrogenicity, personal injury insurance, systems

## Abstract

Functional Neurological Disorder (FND) is a disabling neuropsychiatric condition marked by motor/sensory symptoms not explained by neurological illness. Perceptions of medically unexplained disability, such as FND, are shaped by their social contexts. For example, the introduction of workmen’s compensation legislation (late 19c) prompted labels such as ‘compensation neurosis’. Since the mid 2000s, a biopsychosocial understanding of FND has developed, offering a more nuanced lens through which to examine FND in the personal injury compensation context. This review journeys through historical perceptions of FND in this setting, from its conflation with malingering to its eventual distinction from feigning, before examining how features of compensation environments, such as their legal and administrative structures, are etiologically relevant to FND. We explore how increased understanding of biopsychosocial drivers and prognostic indicators in FND, alongside ‘rule-in’ criteria for early diagnosis, may reduce iatrogenic harm. We also examine whether the biomedical foundations of social insurance programs align with the defining features of the illness. The review highlights the significant contextual relevance of personal injury compensation to recovery outcomes in people who develop FND after physical injury; primarily in an Australian context, but also internationally. Opportunities to more closely align compensation frameworks with the evolved clinical understanding of FND are offered to mitigate against iatrogenic impacts, along with insights to guide future research.

## Introduction: FND mechanisms and the significance of social contexts

Conceptualisations of medically unexplained symptoms (MUS) have been dependent on social contexts, once having been considered supernatural phenomena or witchcraft, in keeping with the dominant religious worldview and medical knowledge (1) of medieval times (2). The scientific pursuit of understanding the medically unexplained disability associated with Functional Neurological Disorder (FND) has revived over the last two decades (3). Notoriously described in Plato’s *Timaeus* (91c-d) as a consequence of a frustrated, ‘unfruitful’ womb (4), history is littered with non-neurobiological explanations of FND, such as blocked ‘life forces’ (1) and sperm retention (5). FND has only recently been associated with neurobiological mechanisms and evidenced by neuroimaging (6). Also central to its modern understanding is its biopsychosocial basis (7); sensorimotor, cognitive and affective processes interact in complex ways with an individual’s socio-environmental context to precipitate and maintain symptoms (8). Bayesian theories conceptualise the integration of physiological (motor sensory), psychological (e.g., illness beliefs) and social factors (e.g., cultural beliefs) (9): these emphasise a mismatch between prior beliefs (over-weighted in FND) and the processing of sensory evidence (under-weighted in FND), mediated by cognition (attention), emotion and socio-cultural factors, which fail to correct aberrant motor predictions (9).

Fundamentally, it is hypothesized that this disorder of motor prediction, planning and agency associated with abnormal attentional focus on the affected body part, is influenced by illness expectations (10), past experiences and beliefs (11). The influence of beliefs is observed, for example, where lay expectations of symptoms associated with a particular disorder shape symptom expression (12, 13). The significance of socio-cultural influences on physical symptom expression has been observed in many contexts. The First World War produced multiple cases of MUS termed ‘shell shock’ or ‘war neurosis’ (14), often resembling soldiers’ exposure to the physical injuries of combat (e.g., injuries to the spinal cord or the visual cortex) (15), resulting in functional paraplegia or blindness (14). More recently, mass hysterical events amongst students involving the rapid spread of illness attributed to an innocuous odour reinforce social influence on functional symptoms (16). Despite theoretical recognition of the significance of social contexts, a recent systematic review of biopsychosocial prognostic indicators in FND (17) highlighted the lack of empirical enquiry into their influence on FND recovery.

The potential for ‘shell shock’ to allow avoidance of combat fuelled suspicions of feigning (18). In less extreme circumstances, the benefits that disability provides within a personal injury insurance context may be considered in the assessment of its validity. With the boundaries between FND, factitious disorder and malingering historically blurred (19), it is unsurprising that conscious and unconscious motivations continue to be questioned. Personal injury insurance creates a social context with a high potential for external reward. Compensation is often payable via statutory benefits (e.g., income replacement, medical expenses, compensation for permanent impairment) (20) and/or court-awarded common law damages for pain, suffering and pecuniary loss (21, pg. 146). FND may be prevalent, given its potential development following injury, or the surgical treatment of injury (22). Disability is shaped by social and structural factors (23), and the modern understanding of FND prompts the question of whether biomedically based social infrastructure, such as government social insurance programs that respond to disability, injury, and unemployment (24), is well suited to this complex condition. Asking this question could inform system modifications to better support functional recovery in this population.

This review first describes historical constructions of MUS, as situated within personal injury compensation globally. It then discusses the disparity in the pace of development of biopsychosocial models of FND and application of this knowledge within personal injury medico-legal contexts. This is followed by a discussion of how outdated clinical practices and legal and administrative principles combine to create a harmful reinforcing feedback loop. The opportunity to modernise personal injury insurance schemes based on contemporary FND models, including a supporting research agenda, is then presented.

### Historical perspectives on medically unexplained symptoms within personal injury insurance

Physical injury compensation dates to the beginnings of recorded history, with the use of money to recompense documented in ancient Sumeria (2050 BC) (25). This social value persists, with social insurance having proliferated globally (24) from its modern foundation in Prussia in 1871 (26). Medically unexplained presentations converged with personal injury compensation during the industrial revolution. When railway travel proliferated in mid-nineteenth century Britain, the condition known as ‘railway spine’, in which somatic symptoms persisted in the absence of substantiated physical injury from railroad accidents (27), attracted competing explanations. Initially, train accidents were thought to subject the spinal cord to structural damage from ‘spinal concussion’ (28), resulting in disability amongst railway workers (29) including ‘weakened intelligence, convulsions and loss of generative power’ (30). Organic explanations gave way to functional theories of ‘psychic shock’ and ‘nervous exhaustion’ toward the end of the century (28). Charcot considered the pathogeny of railway spine to have neurological and psychological components (31), before being reinterpreted as increasingly psychogenic (32). British common law allowed passengers involved in collisions with prolonged disability to seek compensation (27), with ‘railway spine’ central to litigated cases (33). Increased occupational injuries involving significant forces (34) added to the impetus for ‘workmen’s compensation’ laws late in the century (26).

The concept of ‘railway spine’ or ‘accident neurosis’ (a transnational occurrence) (27) quickly became recognised as a partially legal phenomenon attributed to both ‘untoward consequences of frightening occurrences, as well as legal compensation’ (35). The non-anatomical and unexpectedly prolonged presentations prompted terms such as ‘compensation neurosis’ (34) and ‘litigation neurosis’ and even the loquacious ‘social iatrogenesis for disease production by well-intended social programs’ (36). Such presentations were thought to consciously or unconsciously allow a dignified exit from demanding employment (34). The original description of compensation neurosis in 1946 was “a state of mind, born out of fear, kept alive by avarice, stimulated by lawyers, and cured by a verdict” (37, pg. 19). This explanation has been criticised given the failure to consistently observe recovery following litigation settlement (38). Socially, these terms held motivational connotations, but they were not part of the recognised medical nomenclature (34).

In 1979 (39), financial gain became inextricable with unexplained post-traumatic disorders, when ‘compensation neurosis’ was used synonymously with ‘factitious disorder’ in ICD-9 (40) and ICD-10 (41). A more recent conceptualisation by Hall and Hall of ‘compensation neurosis’ (which has never featured as a formal category in any version of the DSM) (36) was as an ‘exaggeration of symptoms that occur as a result of the unique stressor of seeking legally awarded compensation’ (36). They considered it to be not necessarily deliberate, with internal motivators being the greatest driver [e.g., an individual’s concern with being accused of fraudulent behaviour upon recovery, a desire to maintain the preferred change to family/social roles afforded by the impairment, and awareness of the vested interests of others such as family and lawyers (36)]. ‘Compensation neurosis’ therefore was a product of unwarranted illness suggestion (e.g., by lawyers and family), stressful claims processes, its impact on personality vulnerabilities, and the pursuit of justice (36), being differentiated from malingering and factitious disorder by the type of incentives and the absence of conscious deception. This evolution away from ‘avarice’ as a motivator reflects the developing understanding of the complex psychosocial interplay.

### Biopsychosocial advances and the disability-personal-injury-insurance nexus

Since the mid 2000s, examination of biopsychosocial drivers in FND (6, 42–44) has become increasingly nuanced. Hallett et al. (8) proposed that FND symptoms are shaped by interactions between internal predictions and external contexts, where lay expectations, illness beliefs and symptom-attention, (each influenced by socio-cultural factors), modulate FND symptoms (45). For example, benign bodily sensations from a car accident can be misinterpreted (in accordance with lay expectations and beliefs) and immediately result in expected symptoms (10) which reinforce beliefs about their seriousness (46). Sensitivity to entrenched cognitive models informed by prior experiences might explain the amplified experience to sensations in FND (10). Such cognitive models may be reinforced by social factors such as exposure to others/family members with illness/injuries, seen commonly in people with FND (10). The scientific recognition that neurobiological dysfunction in FND is reversible and sensitive to the environment, supports that FND symptoms can be modified for better or worse through social contexts (7).

Whilst FND has been firmly situated within a biopsychosocial paradigm, a mismatch remains between scientific and societal understandings of this under-recognised disorder (47). For example, personal injury insurance schemes have fallen behind advances in the clinical understanding of FND, creating systemic recovery barriers (48). Relevant to this discussion is administrative burden theory (49), which conceptualises the ‘costs’ for individuals participating in social programs associated with learning (e.g., service navigation), psychology (including stigma and stress from processes to access benefits) and compliance (including meeting evidentiary requirements). In FND, these ‘costs’ could foreseeably worsen symptoms through stress-induced mechanisms central to the condition (50). The requirement to meet medical evidentiary thresholds is inherently problematic given that FND is defined in part by the absence of identifiable pathology (51). Typical sources of stress in accessing social insurance following injury in the general population include legitimacy concerns, difficulty obtaining a diagnosis and accessing treatment (52). These stressors mirror difficulties frequently encountered in FND (53). Hence, it is possible that there is a compounding effect of insurance-related stressors for people who develop FND. What once was suspiciously seen as a coincidental resolution of symptoms following legal settlement in ‘compensation neurosis’ might actually be accounted for by the concomitant cessation of stress from processes (36).

### Lost in translation: underuse of modern diagnostic criteria and iatrogenic care

A demarcation between MUS and conscious deception appeared in DSM-III, in which the diagnostic criteria for ‘conversion disorder’ required the exclusion of factitious disorder (54). Specifically, a delineation was made on the basis of intentionality and motivation, with conversion disorder exonerated from an intent to deceive (55). Further diagnostic clarity was achieved in 2013, when DSM-5 (51) ushered in the era of ‘positive physical signs’ for an FND diagnosis, and removed the need to exclude factitious disorder. FND was no longer an exclusionary diagnosis that likely underestimated prevalence (56); the ‘rule-in’ approach providing a mechanism to expedite diagnosis without the sole reliance on the exclusion of organic pathologies. Although these tests may not differentiate FND from voluntary deception (57), this important development holds promise for timelier FND diagnosis, and therefore improved recovery outcomes (58). For example, the explanation of an FND diagnosis itself can be therapeutic, leading to the immediate cessation of non-epileptic seizures in 32% of cases (59).

Despite this apparent clarity, clinicians seem reluctant to let go of the diagnosis of FND by exclusion. A 2021 article (60) evidenced that just over half of neurologists surveyed still considered FND to be an exclusionary diagnosis. A retrospective review of referrals to a specialist functional neurology clinic discovered only one-quarter had made reference to rule-in signs (61). Reasons for reluctance to depend on positive signs include fear of missing organic pathology (62), despite a 4% false negative rate (63). Evidence also supports that only 4% of medical practitioners recognised positive physical signs, and the risk of harms from under-diagnosing FND was under-recognised by half of medical practitioners (64). The timeliness of specialist assessment is impacted (65) by the efficiency of referral by primary care physicians, however as few as 14% of Australian general practitioners described a ‘good’ knowledge of FND (66). At a systems level, FND is likely to be under-coded in the healthcare system (56, 67). The general diagnostic ambiguity associated with FND has the potential to adversely shape illness beliefs (such as there being an organic cause), fuelling anxiety and exacerbating symptoms (46). Unsurprisingly, the average delay between functional symptom onset and the first psychotherapeutic or psychiatric treatment is 6–25 years (68–70) with poorer outcomes demonstrated when the condition has been present for over one year (71). In the context of an insurance claim, clinicians’ views may be biased toward an organic cause when there has been a history of mechanical insult to the body.

The avoidance of iatrogenic harm in FND has been highlighted (72), with common consequences including unnecessary medical treatment and stigma-based psychological harm, including patients feeling dismissed (48). Iatrogenesis associated with delayed or inappropriate treatment relates to chronicity and deconditioning (48). Physiologically, there may be maladaptive neural plasticity (73) such as learned non-use, trophic changes (74), muscular atrophy, and soft tissue contracture (thwarting later attempts to improve function). Interventions that may be routine components of musculoskeletal rehabilitation (e.g., gait aid prescription) should proceed with caution in FND (74). The development of maladaptive movement patterns has been recognised in Occupational Consensus guidelines for FND, which recommend short-term equipment use only, with a clear progression plan (74). Psychologically, prolonged illness duration reduces self-efficacy (75) and predisposes to psychopathology (76). Socially, family dynamics and roles shift, relationships become strained, with impacts felt across diverse life domains (77). Within a compensation context, there may be the particular risk of supplier-induced healthcare over-utilisation (78), perpetuated by an individual’s engagement in medical validation (79).

### FND illness features v. personal injury legal doctrines: the inherent incompatibility

Further iatrogenic potential arises when a comparatively nebulous condition such as FND presents within a medico-legal setting, as approximately one-fifth of patients with FND experience (71). Firstly, it can be difficult to establish a causal nexus between the FND presentation and the original compensable injury, when conventional medical tests fail to find the pathology accounting for the prolonged disability. Schemes differ between jurisdictions, however those derived from the English common-law tradition (e.g., Canada, Australia and New Zealand) share commonalities (80, 81). For example the compensable event may only be required to be ‘a cause’ of impairment, regardless of an individual’s pre-existing state (82, 83). This complicates the assessment of liability at any point in time because whilst an accident may be the catalyst for symptom onset, other factors contribute to their maintenance (8).

Causality can also appear tenuous due to FND temporal features. For example, there may be a significant latency between the precipitating injury/trauma and the onset of FND (10, 84). Delays in symptom onset also mean that a subjective history not reviewed in the context of contemporaneous objective medical records can create a false narrative about the organic injuries. For example, contemporaneous records can assist in differentiating stress-induced dissociation, erroneously labelled as ‘loss of consciousness’ from a head injury (46). The emergence of FND symptoms during the rehabilitation of an injury (85) might also challenge a causal relationship, but may be understood by the nature of the treatment context; given that rehabilitation increases the attention afforded to the injured part through attempts to progressively return its use in functional activities, it is conceivable that attention-based mechanisms underlying symptom production (9) activate.

The observation that disability is inversely proportional to the severity of physical injury has long raised suspicion (34). This has been described as an unconscious ‘compensatory relationship’ where intangible injury is more likely to produce reactive anxiety or depression that is expressed physically (34). This supports the notion that FND symptoms are emotional expressions influenced by prior experience and augmented by social contexts (86). The high prevalence of adverse life events prior to FND onset (87) might plausibly contribute to an unconscious motivation to seek restorative justice from the ‘at-fault party’, possibly extending beyond the accident itself. This is supported by the finding that adverse events preceding FND onset are particularly likely to be those offering secondary gain (88). In medico-legal contexts, clinicians already report reduced confidence in their judgment compared to clinical practice, in addition to the increased tendency to request further information (89). This may be heightened amidst the ambiguity of FND, with the potential to add to delays in diagnosis and treatment.

Once liability is established, the extent of harm attributable to the defendant is assessed. This occurs following the determination of clinical stability. There is Australian evidence that solicitors over-estimate the time for stability attainment, thereby delaying the issuing of a writ (90) and protracting stressful legal processes. In FND, ‘stability’ is conceptually complicated due to its waxing-waning nature (91). One key cause of fluctuating presentations in FND is stress-induced exacerbations. This association has been supported by neuroimaging data suggestive of abnormal limbic-motor communication with emotional stimuli (86). It is suggested that heightened emotion increases body-focussed attention and the expected certainty of abnormal symptoms (influenced by prior beliefs), which exacerbates them (9).

Another source of symptom fluctuation in FND is improvement with distraction, which is theorised to alter attentional mechanisms and interrupt automatic movement processes (92). That distraction improves symptoms conflicts with the legal focus on the assessment of impairments of body structure and function for the determination of financial compensation. Practically, this means the recounting of impairments to lawyers, employers, family/friends, treating health and medico-legal professionals may worsen symptoms (36). It is also possible that the constant need to reaffirm impairment as accident-related fuels the internal processes hypothesised to reinforce disability described in ‘compensation neurosis’. It is unsurprising that there is a stronger association between compensation/legal processes and health outcomes, than with the severity of the initial injury (93).

The apportionment of harm by the defendant is complicated by pre-existing psychosocial stressors common in FND (94) and their entanglement with common accident-related sequalae (e.g., PTSD, depression and anxiety) (95). A common legal response to pre-existing vulnerabilities is the ‘egg-shell skull plaintiff’ rule, applied across common law jurisdictions (96). This mandates that the “defendant takes the victim as found”. Practically, this means that the plaintiff’s pre-existing vulnerability to injury does not reduce the defendant’s liability for harm to which they substantially contributed (97). Therefore, the defendant may be found liable for harm that was not reasonably foreseeable, because of the individual’s existing fragility, so that the ensuing impairment and disability, which might not have occurred in a less fragile individual, must be compensated. It has been argued (96) that egg-shell-skull type liability encourages suboptimal behaviour such as failure to ‘self-protect against losses’. It has also been warned (97) that this doctrine conflates ‘subsequence’ with ‘consequence’, ignoring that psychological symptoms at any given time are produced by complex biopsychosocial interactions.

We argue that the ongoing acceptance of liability by the defendant for the natural course of a pre-existing vulnerability and associated provision of funded supports is disempowering for FND recovery. This is supported by evidence of ‘utilisation hazard’, where patients overuse insurance-funded medical services at no financial costs to themselves (98). Over-medicalisation in FND carries its own iatrogenic risks (48) including sick-role entrenchment (99) and the reinforcement of symptomatology (10). The risk of disempowerment may be enhanced in FND due to the propensity for comorbid personality disorders, such as dependent-type, and its association with worse health outcomes and increased healthcare utilisation (100). To mitigate the possibility of failure to self-protect against losses, Calandrillo (96) suggests that defendants should only be liable for the ‘reasonable foreseeable scope of victims’ injuries’. This approach is more aligned with the ‘crumbling skull’ rule (83) which recognises the progression of an existing deteriorating condition and spares the defendant from damages associated with its expected course. Given the potential for iatrogenicity, it is reasonable to suggest that such clear boundaries to liability might be in an individual’s best recovery interests. In a stress-sensitive condition such as FND, where it is expected that intermittent support may be required throughout the lifespan (mirroring periodic exposures to life stressors), symptomatology may come to reflect the natural course of the condition, and the effects of access to funding need to be considered.

A balance therefore must be struck between equitable compensation and inadvertent harm through ‘over-compensation’. For people vulnerable to developing FND, advantages of access to compensation include faster access to specialist assessment and treatment, wage replacement and return to work support. Harm however may occur if a subsequent injury/condition becomes causally linked to the compensable event, resulting in surgery where a psychosomatic basis predominates (101), and unnecessary, costly procedures with life-changing consequences [e.g., limb amputation (101)]. This risk is heightened given that multiple MUS are common in FND, with up to 57% of people with functional seizures having at least one other MUS (102). These MUS may present spontaneously in predisposed individuals, regardless of an accident (46). Given the diverse manifestations of FND (51), funding might be sought for an array of medical treatments and surgeries traditionally reserved for organic injuries (103). The merit of such interventions according to a value-based health care paradigm (104) may not determine their utilisation; unconscious resistance to continuing treatment options that improve symptoms may exist if those symptoms and associated monetary benefits offer escape from life stressors (105), including those that are work-related (88) and which may have triggered the compensation claim.

### The biomedical legacy of social insurance programs and its mismatch with FND

The discrepancy between the variability of FND symptomatology and the expected recovery course of the original compensable injury is problematic. Many personal injury insurance claims are musculoskeletal (106), where a linear, unidirectional recovery trajectories might typically be expected. Traditional models of injury rehabilitation, consistent with a biomedical approach, have reinforced this (107). It is unsurprising that compensation laws have been crafted and administered with the majority experience in mind. For example, tests of recovery used to determine access to income and other support services at mandated points in time reflect an expectation of continual recovery and progressive independence from the scheme. This is at odds with FND’s often remitting, relapsing nature. The biomedical foundations of social insurance schemes may therefore be ill-suited to neuropsychiatric conditions with fluctuating presentations, such as FND.

The expectation of linearity and functional consistency enshrined in the insurance assessment process can, in FND, erode the interpretation of conventional methods of ‘evidence’ collection. The variable nature of FND may appear at odds with the course of many organic conditions, and potentially adversely affect the perceived authenticity of the presentation (108). This may be apparent in the use of surveillance in FND. There may be a misunderstood discrepancy between function observed covertly on a ‘good day’ when distracted and unaware of observation, and patient-reported ‘typical’ accounts (46). The conclusion that the witnessed footage indicates a work capacity or the absence of the need for funded supports, and the communication of a decision on this basis, could plausibly fuel the hypothesised attention and emotion-mediated causal pathways in FND with the potential to exacerbate symptomatology. Unsurprisingly, it has been suggested that the determination of the compensability of FND symptoms may rest more with the plaintiff’s perceived credibility than clinical factors (65).

Historically, psychiatric sequalae of accidents at work or on the roads has been minimised and considered to apply to the minority, which has posed a problem for ensuring adequate treatment and rehabilitation (109). The focus on musculoskeletal recovery has also been evident in commentary about impairment guidelines for psychiatric disability, which have been described as ‘simplistic and inadequate’ (109). This is unsurprising given the historically restrictive legal doctrines imposed on the assessment of psychiatric liability (110) compared to physical injuries (111). Another legacy of the biomedical emphasis is the erroneous assumption that physical injury type and severity alone correlate with the degree of functional impairment. Waddell’s seminal work (112) mathematically challenged this approach and quantified the lack of correlation between structural impairment, pain and disability. Studies continue to demonstrate that physical injury type and severity alone do not predict functional outcomes (113).

## Future directions

This review describes the interplay of biopsychosocial factors in FND, their intersection with personal injury insurance factors, and how this intersection contributes to increased vulnerability to poor outcomes for people who develop the condition. The understanding of the complex biopsychosocial determinants of FND has progressed, and validity has been afforded to its neurobiological basis. Emerging insights based on FND etiology provide the impetus to further interrogate the complex interactions between FND and personal injury insurance environments. To support this, a concerted effort to accurately capture FND prevalence across health and compensation systems is required. Initial research should quantify and compare outcomes in people matched on compensable physical injury type, when FND does and does not develop. Contextualisation of this data, and the exploration of outcome heterogeneity, should occur by qualitatively exploring clinicians’ and patients’ perceptions of influential system factors, to then inform and test system-based prediction models. Modifiable elements of compensation systems influential to recovery in people who develop FND, and other conditions, can then be targeted to improve health outcomes (114).
